# Identification of Prognostic Alternative Splicing Signature in Breast Carcinoma

**DOI:** 10.3389/fgene.2019.00278

**Published:** 2019-03-28

**Authors:** Dong Zhang, Yi Duan, Jinjing Cun, Qifeng Yang

**Affiliations:** ^1^Department of Breast Surgery, Qilu Hospital, Shandong University, Jinan, China; ^2^Department of Pathology Tissue Bank, Qilu Hospital, Shandong University, Jinan, China

**Keywords:** bioinformatic analysis, alternative splicing, breast carcinoma, prognostic model, nomogram, gene set enrichment analysis, gene set variation analysis

## Abstract

**Background:**

Increasing evidence indicated a close relationship between aberrant splicing variants and carcinoma, whereas comprehensive analysis of prognostic alternative splicing (AS) profiling in breast cancer (BRCA) is lacking and largely unknown.

**Methods:**

RNA-seq data and corresponding clinical information of BRCA patients were obtained and integrated from The Cancer Genome Atlas (TCGA). Then SpliceSeq software was used to assess seven AS types and calculate the Percent Spliced In (PSI) value. Univariate followed by stepwise multivariate Cox regression analyses identified survival associated AS events and constructed the AS signature, which were further sent for enrichment analysis, respectively. Besides, the splicing correlation network was constructed. Additionally, nomogram incorporating AS signature and clinicopathological characteristics was developed and its efficacy was evaluated with respect to discrimination, calibration and clinical utility.

**Results:**

A total of 45,421 AS events were detected, among which 3071 events were found associated with overall survival (OS) after strict filtering. Parent genes of these prognostic events were involved in BRCA-related processes including NF-kappaB and HIF-1 signaling pathway. Besides, the final prognostic signature built with 20 AS events performed well with an area under the curve (AUC) of receiver operating characteristic (ROC) curve up to 0.957 for 5 years. And gene set enrichment analysis (GSEA) also confirmed the candidate 20 AS events contributed to progression of BRCA. Moreover, the nomogram that incorporated 20-AS-event-based classifier, age, pathological stage and Her-2 status showed good calibration and moderate discrimination, with C-index of 0.883 (95% CI, 0.844–0.921). Decision curve analysis (DCA) confirmed more benefit was added to survival prediction with our nomogram, especially in 5 or 8 years with threshold probability up to 80%. Finally, splicing correlation network revealed an obvious regulatory pattern of prognostic splicing factors (SF) in BRCA.

**Conclusion:**

This study provided a systematic portrait of survival-associated AS events involved in BRCA and further presented a AS-clinicopathological nomogram, which could be conveniently used to assist the individualized prediction of long-term survival probability for BRCA patients. And a series of bioinformatic analysis provided a promising perspective for further uncovering the underlying mechanisms of AS events and validating therapeutic targets for BRCA.

## Introduction

Breast cancer (BRCA) is one of the most common malignant tumors and leading cause of cancer-associated death in women worldwide (DeSantis et al., 2014). As was reported, a decrease in long-term survival was brought about from 90 to 5% once distant metastasis to other organs or recurrence occurred, leading to poor prognosis of BRCA patients (Greenberg et al., 1996). In addition, the heterogeneity and complexity of BRCA introduced a challenge in comprehensively understanding of BRCA carcinogenesis, progression, invasion, and metastasis with traditional clinicopathological and molecular markers (Cancer Genome Atlas Network, 2012), contributing to serious delay on early diagnosis. Therefore, it was sorely required to excavate novel biomarkers with high accuracy of assessment of diagnosis and prognosis in BRCA patients.

Over the last decades, intensive efforts have been made to explore the underlying mechanisms of BRCA and further facilitated the identification of prognostic markers and even therapeutic targets (Cancer Genome Atlas Network, 2012; Sparano et al., 2018). It was widely accepted that dysregulation of gene expression (Cruz et al., 2018), copy number variation (Long et al., 2013), and DNA methylation (Visvanathan et al., 2017) were involved in the initiation and progression of BRCA. However, these studies, although with promising results, were mainly confined to transcriptional level while systematic analysis of splicing variant on transcript architecture is always ignored.

Alternative splicing (AS), as a post-transcriptional regulatory mechanism, hold the largest potential to generate varied isoforms among nearly 90% of human protein-coding genes (Baralle and Giudice, 2017). Substantially, precursor mRNA can be spliced into different arrangements to produce structurally and functionally protein variants via removal of intronic regions, selective inclusion or exclusion of specific exons within multi-exon genes (Kelemen et al., 2013), which further contributed to the proteome diversity and phenotypic complexity (Leoni et al., 2011). Recently, due to the technical improvement of high-throughput sequencing (Foulkes et al., 2003), the correlation between particular AS events and several cancer-related hallmarks such as epithelial-mesenchymal transition (Pradella et al., 2017), anti-apoptosis (Kedzierska and Piekielko-Witkowska, 2017), migration and invasion (Leggere et al., 2016) has been gradually recognized and validated. Hence, identification of specific AS events is much more precise and concrete than restriction to transcriptome level in terms of clinical application as prognostic and predictive biomarkers as well as therapeutic targets in BRCA (Johnson et al., 2015; Kladi-Skandali et al., 2018).

It was widely accepted that pre-mRNA splicing can be regulated by both cis-regulating sequences and trans-acting factors. According to different locations and special effect on the usage of a splice site, cis-regulatory sequences could be classified into exonic splicing enhancers, exonic splicing silencers, intronic splicing enhancers and intronic splicing silencers, which determined their affinities to cognate splicing factors (SFs). However, trans-acting factors, including members of well characterized Ser/Arg-rich and heterogeneous nuclear ribonucleoprotein (hnRNP) protein families, as well as tissue-specific factors, function through binding to exonic splicing enhancers and silencers, further leading to the activation or inhibition of specific splice sites (Kornblihtt et al., 2013). Interestingly, many of the trans-acting SFs can act in both ways based on the sequence and position of their specific target site within the genomic region of pre-mRNA (Ule et al., 2006). Moreover, it was proved that aberrant AS events is orchestrated by the dysregulation of SFs (Anczukow and Krainer, 2016). SFs influence splice site selection of splicing regulatory complex called spliceosome via binding pre-mRNA at exonic splicing enhancers or silencers (Shi, 2017). Thus, it is also imperative to seek potential regulatory relationships between SFs and AS events in BRCA.

In our study, comprehensive profiling of genome-wide alternative splicing events was performed in a strictly screened BRCA cohort from The Cancer Genome Atlas (TCGA) database. A robust prognostic signature based on AS events was constructed in BRCA, and GSEA results accompanied with prognostic SF-AS network also revealed the underlying mechanism at respect of BRCA prognosis. Furthermore, our study made the first attempt to establish a prognostic nomogram for BRCA based on AS data, which could be further applied in clinic as prognosis element and prediction for long-term survival of individualized BRCA patient.

## Materials and Methods

### Data Curation Process

The BRCA dataset, including level 3 RNA sequencing data and corresponding clinical information were downloaded and integrated via TCGAbiolinks from TCGA data portal^1^ (Colaprico et al., 2016). To generate the AS profiling for each BRCA patient, SpliceSeq, a Java application that unambiguously quantify the inclusion level of each exon and splice junction, was used to assess the RNA splicing patterns for each sample in BRCA dataset and calculate the percent spliced-in index (PSI) value, which represented the transcript ratio of parent gene to seven types of AS events (Ryan et al., 2012). To generate as reliable a set of AS events as possible, we implemented a stringent filter that percentage of Samples with PSI Value was not less than 75. The inclusion criteria were as follows: (1) female; (2) a histological diagnosis of breast carcinoma; (3) patients who didn’t receive neoadjuvant treatment; (4) patients with complete and definitive clinical features including age, histologic classification, pathological stage, T stage, N stage, regional lymph nodes involvement, estrogen receptor (ER), progesterone receptor (PR), and human epidermal growth factor receptor-2 (Her-2) status; (5) patients were still alive at least 30 days after initial pathologic diagnosis of BRCA; (6) patients with corresponding RNA-seq splicing variant data. Patients with unknown or ambiguous information were excluded. Besides, the immunohistochemical (IHC) status and result of fluorescence *in situ* hybridization (FISH) were both taken into account for defining the accurate Her-2 status of patients (Xu et al., 2018). As a result, 645 patients were included in our study cohort.

In addition, each AS event was assigned a unique annotation via combining the splicing type, ID number in SpliceSeq database and matched gene symbol together to describe them precisely. For instance, in the annotation term “ME_HLCS_ID_96019,” mutually exclusive exons (ME) represented the splicing type, ID_96019 stood for the specific ID of splicing variant and HLCS was the corresponding gene symbol.

### Identification of Survival Associated AS Events, Functional Enrichment Analysis, and Gene Set Variation Analysis (GSVA)

For each type of AS events, BRCA cohort were divided into two groups by a median cut of PSI value. Univariate Cox proportional hazard regression analysis was performed to identify overall survival (OS) associated AS events with *P* < 0.05. Upset plot was drawn to display the interactive sets between seven types of OS-associated AS events with UpsetR package in R (version 1.3.2). Then the parent genes of OS-associated AS events were sent for functional enrichment analysis via clusterProfiler package (version 3.10.0) (Yu et al., 2012). Gene Ontology (GO) terms and Kyoto Encyclopedia of Genes and Genomes (KEGG) pathways with false discovery rate (FDR) less than 0.05 were considered enriched significantly. Then the top significant pathways in KEGG and each GO category including cellular component (CC), molecular function (MF), and biological process (BP) were visualized with bubble diagram, respectively.

Gene set variation analysis (GSVA) was a non-parametric and unsupervised algorithm estimating variation of pathway activity over heterogeneous samples by yielding sample-wise enrichment scores. Moreover, GSVA was applied on the parent genes of OS-associated AS events using GSVA package (version 1.30.0) to further identify significantly enriched set of GO and Canonical pathways (KEGG, Reactome, and BioCarta pathway database) in BRCA tissue, which were downloaded from Molecular Signatures Database (MSigDB)^2^ (Hanzelmann et al., 2013; Liberzon et al., 2015). Then we use limma package to detect up-regulated gene sets in tumor tissue compared to adjacency normal samples (Ritchie et al., 2015), setting logFC > 0.4 and FDR < 0.05 as cutoff value for GO terms and pathway sets.

### Construction of the Prognostic Signature Using AS Events for BRCA Patients

The most significant survival-associated AS events in each splicing type were selected as candidates to fit multivariate Cox hazard regression for BRCA cohort, respectively. Backward stepwise variable selection was applied to avoid model overfitting by reducing Akaike’s information criterion (AIC) to a minimum as the stopping rule. Taken the differential and independent pattern of seven AS types on post-transcriptional modification into consideration, we gathered the AS events identified above together to fit another multivariate Cox regression. To make the final model more practical and parsimonious, forward stepwise approach which began with null model was used to find a minimal set of AS events (Zhang, 2016b).

Then risk scores for each prognostic signature were calculated based on the sum of products of PSI values of identified AS events and corresponding coefficients generated from Cox model, respectively. BRCA patients were divided into high- and low-risk subgroups by median risk score for fitting Kaplan–Meier survival analysis to further validate whether they went through diametrically distinct prognosis. And the predictive accuracy of each prognostic signature was accessed by calculating Uno’s inverse-probability of censoring weighting estimation of dynamic time-dependent receiver-operator characteristic (ROC) area under the curve (AUC) values (time spanning from 3 to 8 years) with timeROC package (version 0.3), according to the method proposed by Blanche et al. (2013). The significance differences of AUCs over time between final AS signature and models built by one type of AS events were further evaluated with plotAUCcurveDiff function.

In order to validate independent predictive power of AS event-based predictive model from clinicopathological factors in BRCA cohort such as age, pathological stage, ER status, PR status, Her-2 status, the stratified Cox proportional hazard analysis was constructed (Zhang, 2016a).

### Gene Set Enrichment Analysis (GSEA) for the AS Event-Based Classifier

To explore the involving pathway of AS events included in final predictive model in terms of tumorigenesis and progression, gene set enrichment analysis (GSEA) was performed with the JAVA program using the Canonical pathways gene set access from MSigDB (Liberzon et al., 2015). And genes were ranked on the basis of differential significance between the high- and low- risk subgroups, which were classified by the AS event-based classifier in the BRCA cohort. Gene sets with nominal *P* < 0.05 and FDR < 0.1 after performing 1,000 permutations were considered significantly enriched.

### Development and Apparent Performance of a AS-Clinicopathologic Nomogram

To formulate a nomogram for better prediction of the individualized survival rate of BRCA patients, multivariable Cox regression analysis combining the 20-AS-event-based classifier with all informative clinicopathologic variables described above was performed using the rms package (version 5.1.2) (Zhang and Kattan, 2017). And variables included in the final nomogram were determined by a backward stepwise variable selection with the Akaike information criterion (AIC).

Calibration curves were plotted to evaluate the predictive accuracy of the final nomogram, and concordance index (C-index) was measure to quantify its discrimination ability with Hmisc package (version 4.1.1). Then the decision curve analysis (DCA) was conducted to estimate clinical utility of the nomogram through quantifying net benefits against a range of threshold probabilities (Rousson and Zumbrunn, 2011; Balachandran et al., 2015).

### Construction of Potential SF-AS Regulatory Network

A list of 67 human SF was extracted from the SpliceAid 2 database^3^ (Supplementary Table S1; Piva et al., 2012). The expression profiles of SFs were obtained from TCGA data portal and normalized by division by size factors with variance Stabilizing Transformation function from DESeq2 package (version 1.22.1) (Love et al., 2014). Univariate Cox regression analysis was conducted to screen survival-associated SFs, and the optimal cut points, which separated high-risk subgroup from low-risk one using the maximally selected rank statistics, were also detected to fit Kaplan–Meier survival analysis for further validation (Hothorn and Zeileis, 2008). Next, Spearman correlation analysis between expression level of OS-associated SFs and PSI values of AS events that were included in construction of each prognostic signature was performed. *P*-values were adjusted by Benjamini and Hochberg (BH) correlation. Then the potential SF-AS regulatory network was generated among the significant correlation pairs (adjusted *p* < 0.05) by Cytoscape (version 3.6.1).

## Results

### Overview of AS Events Profiles in TCGA BRCA Cohort

**Figure 1:**
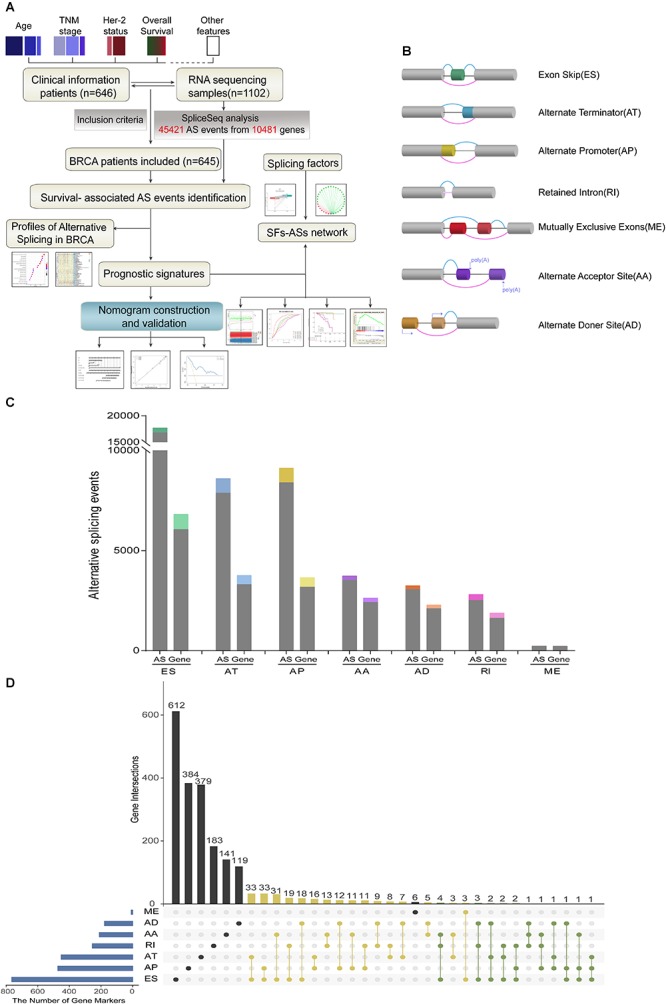
Overview of AS events profiling in BRCA cohort. **(A)** Flowchart for profiling AS of BRCA. **(B)** Illustration for splicing pattern of seven types of AS events, including Exon Skip (ES), Alternate Promoter (AP), Alternate Terminator (AT), Alternate Donor site (AD), Alternate Acceptor site (AA), Mutually Exclusive Exons (ME), and Retained Intron (RI). **(C)** Seven types of AS events and corresponding genes from the 645 BRCA patients were depicted according to classified *P*-value of 0.05. The gray bars represent the prognosis irrelevant AS events and involved genes. The color bars represent the prognostic AS events and parent genes filtered by univariate Cox analyses. **(D)** Upset plot of gene interactions between the seven types of prognosis-related AS events (*n* = 3071). The yellow dot lines represent that one gene could have two types of prognostic AS events while blue dot lines represent the genes which occupied up to three types of survival-associated AS.

Detailed processes of our study design are illustrated in Figure 1A as a flowchart. Integrated mRNA splice variant profiles were curated with detailed AS events data and clinical information of 645 BRCA patients, among which median follow-up period was 16.7 months (range, 1–197 months). AS events were roughly divided into seven types including Exon Skip (ES), Alternate Promoter (AP), Alternate Terminator (AT), Alternate Donor site (AD), Alternate Acceptor site (AA), Mutually Exclusive Exons (ME), and Retained Intron (RI), and their assigned splicing pattern were presented in Figure 1B. By using SpliceSeq, we totally detected 45,421 splicing events in 10,481 genes, comprised of 17,702 ESs in 6812 genes, 8595 ATs in 3755 genes, 9112 APs in 3654 genes, 3731 AAs in 2628 genes, 2802 RIs in 1878 genes, 3246 ADs in 2278 genes and 233 MEs in 227 genes (Figure 1C). These data showed a single gene might have almost four types of AS events on average, which indicated that different arrangement and combination of splicing types held great potential responsible for transcriptome diversity. Besides, ES was the predominant component for it accounted for nearly forty percent of all species of AS events.

### Identification and Functional Enrichment Analysis of Survival-Associated AS Events

For each type of AS events, BRCA patients were dichotomized as low- and high-PSI subgroup based on the median cut of PSI value. Moreover, univariate survival analysis was applied to distinguish AS events of survival associated group (*p* < 0.05) from survival irrelevant ones (*p* > 0.05). And a total of 3071 AS events from 2075 parent genes were identified as candidate prognosis-related AS events, accounting for 6.76% of the total AS events and 11.72% of total patent genes in BRCA, respectively (Figure 1C). With the visualization of Upset plot, one gene could possess up to three AS types, which were all closely related with OS (Figure 1D). For example, AT, AP, and ES event of KCTD7 (green dot line) were all significantly associated with patients’ survival.

**Figure 2:**
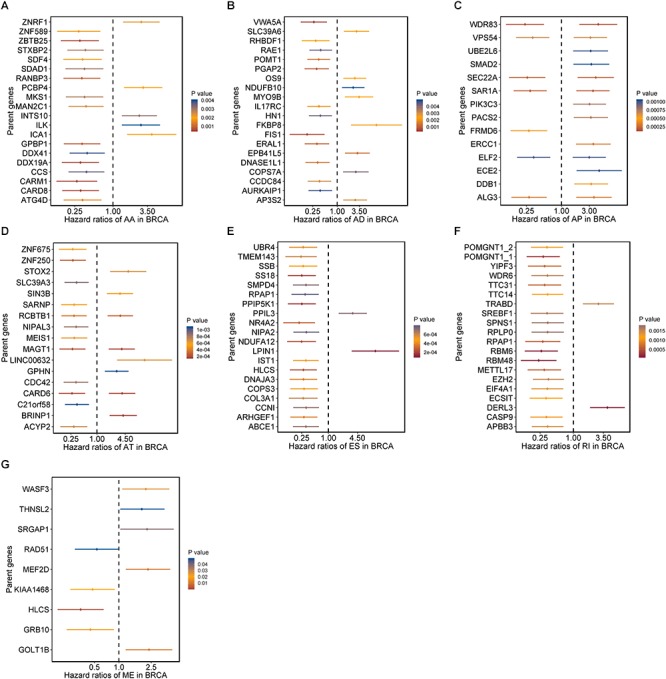
Forrest plots of survival associated AS events in BRCA. **(A–F)** Hazard ratios and 95% confidence intervals of top 20 overall survival associated AA, AD, AP, AT, ES, and RI events, respectively. **(G)** Hazard ratios and 95% confidence intervals of significant OS associated ME events. *P*-values of univariate Cox analyses are indicated by color scale by the side.

Moreover, hazard ratios (HRs) with 95% confidence intervals (95% CI) of top 20 most significant AS events (if available) for each AS type were displayed using forest plot (Figure 2). Obviously, most of these AS events in ES and RI were favorable prognostic elements. Besides, one gene could process AS events that have significantly opposite effect on survival, which would be undetectable if we were merely restricted to transcriptome level.

**Figure 3:**
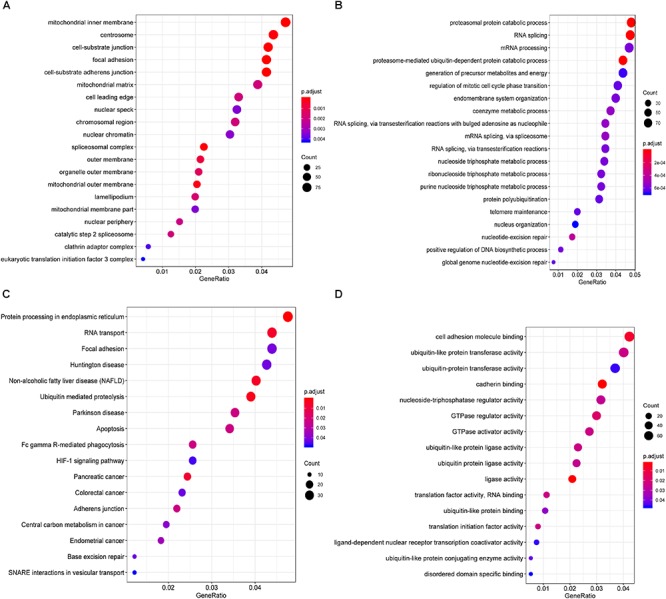
Functional analyses on parent genes from survival-related AS events in BRCA, including GO and KEGG. Top 20 pathways (if available) of GO term in cellular component **(A)** biological processes **(B)** molecular function **(C),** and KEGG pathway **(D)** analyses of genes from survival associated AS events, respectively. The dot size represents the enriched gene number and FDR values are indicated by color scale by the side.

**Figure 4:**
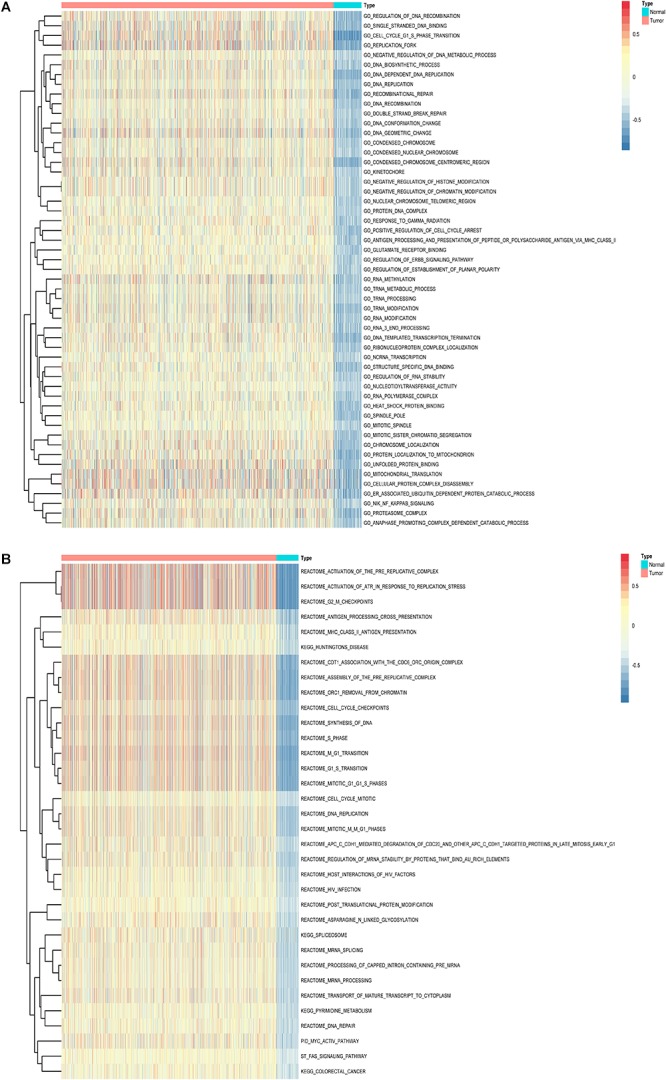
Gene set variation analysis (GSVA) results with hierarchical clustering on parent genes from survival-related AS events in BRCA. **(A,B)** Heatmaps of upregulated differential enrichment results in BRCA tissue through GSVA of GO and Canonical pathways (KEGG, Reactome, and BioCarta pathway database) from MSigDB. Color transition from blue to red indicates the increasing enrichment score of BRCA samples.

Furthermore, to shed light on the potential interference of OS associated AS events and corresponding protein, all parent genes of survival-related AS events in BRCA were further sent for bioinformatic analyses, including GO, KEGG, and GSVA. A total of 84 terms were identified in cellular component (CC) with top significative terms in aspects of cell adhesion, spliceosome complex and mitochondrial content (Figure 3A). Besides, 212 pathways in biological process (BP, Figure 3B) and 17 pathways in molecular function (MF, Figure 3D) were also highlighted, indicating significant difference in terms such as RNA splicing, DNA damage repair, protein ubiquitination as well as purine related metabolic process. Additionally, 17 specific KEGG pathways were confirmed significant and several pathways which reported to be associated with BRCA prognosis were enriched (Figure 3C), including HIF-1 signaling pathway (FDR = 0.0463), ubiquitin mediated proteolysis (FDR = 0.00354) and apoptosis (FDR = 0.0236) related pathways. Top significant enriched terms were displayed in Figure 3. And the detailed information concerning functional enrichment analyses was also included (Supplementary Table S2). To further confirm functionally enriched gene sets in BRCA, GSVA was performed and 53 significantly activated terms in MSigDB C5 GO and 34 significantly upregulated pathways in C2 canonical pathways were identified in tumor samples (Figure 4A,B). Tumor tissue exhibited increased activities in cell proliferation and mRNA splicing which was consistent with GO and KEGG results described above. Moreover, cancer-specific gene sets were also identified with NF-KappaB, ERBB, and Fas signaling pathway upregulated in tumor tissue. Taken together, these results indicated that parent genes of prognostic AS events played crucial roles in biological process of BRCA, contributing to uncover potential modification mechanisms of OS-associated AS events toward protein function.

### Construction of the Prognostic Signature Using AS Events for BRCA Patients

**Figure 5:**
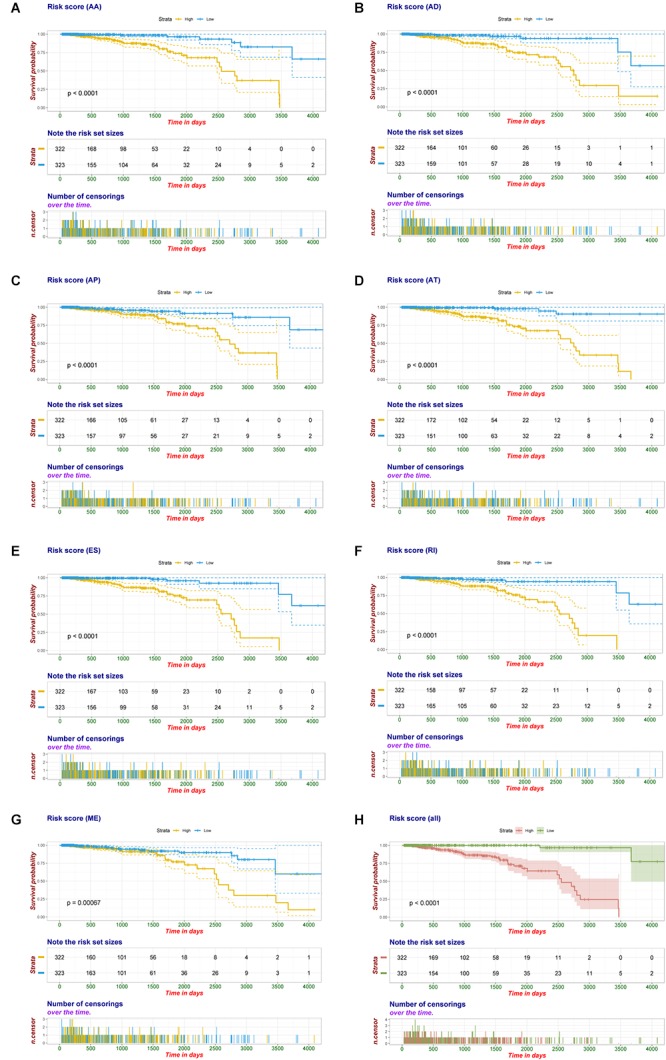
Kaplan-Meier plot of prognostic signature built with AS events for BRCA patients. **(A–G)** Kaplan-Meier plot of prognostic signature built with AA, AD, AP, AT, ES, RI, and ME events, respectively. Yellow line indicates high-risk subgroup while blue line indicates low-risk subgroup. **(H)** Kaplan–Meier curves of final prognostic signature built upon all types of AS events for BRCA patients, in which red line represents high-risk subgroup and green line represents low-risk subgroup.

**Figure 6:**
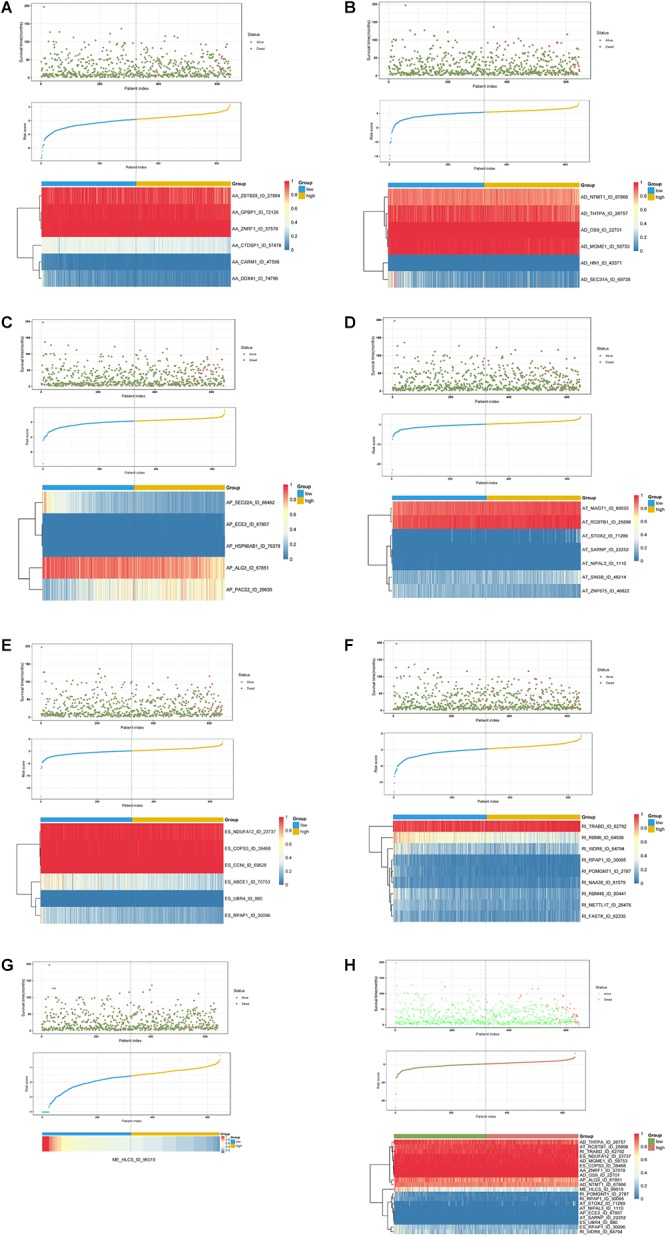
Determination and analysis of the prognostic AS signatures in BRCA cohort. BRCA patients were divided into high- and low-risk subgroups based on the median cut of risk score calculated separately. The upper part of each assembly indicates distribution of patients’ survival status and survival times ranked by risk score, the middle part represents the risk score curve, and the bottom heatmap displays splicing pattern of the AS signature from each AS type or all seven AS types. Color transition from blue to red indicates the increasing PSI score of corresponding AS event from low to high. **(A–G)** Risk score (corresponding to each AS type) calculated and AS signature constructed using each AS type of prognostic splicing events. **(H)** Risk score (all) calculated and final AS signature constructed using all types of prognostic splicing events.

Multivariate Cox regression analyses following backward stepwise selection were applied to the most significant survival-associated AS events in each AS type for BRCA cohort, including AA events (*P* < 0.005), AD events (*P* < 0.01), AP events (*P* < 0.002), AT events (*P* < 0.001), ES events (*P* < 0.001), RI events (*P* < 0.002), or ME events (*P* < 0.05), respectively. Risk score was calculated based on screened AS events in each splicing type, and BRCA patients were stratified into high- and low-risk subgroups based on the median value of risk scores. As presented in Figure 5, the AS signatures constructed with 6 AA events, 6 AD events, 5 AP events, 7 AT events, 6 ES events or 9 RI events all showed great power in distinguishing the two subgroups of different risk patterns (*p* < 0.0001). Thereinto, signature based on only 1 ME event was also able to differentiate between high-risk group and low-risk one in spite of limited data at present (*p* = 0.00067). Then we performed forward stepwise selection on 40 AS events gathered from seven types of AS signatures to simplify the final signature only with 20 AS events, which consisted of 4 AD events, 4 AT events, 4 ES events, 4 RI events, 2 AP events, 1 AA event, and 1 ME event. The detailed information and illustration of these particular AS events are summarized (Table 1 and Supplementary Figure S1). Kaplan-Meier survival analysis of the final signature indicated that there was a notable difference in survival times between two subgroups distinguished by the 20-AS event-based signature (*P* = 8e-13, Figure 5H) with median survival time over 4000 days in the low risk group. The distribution of patients’ survival status and risk score, and the splicing pattern of the AS signature for each AS type or all seven AS types were visualized in Figure 6.

**Table 1:** Detailed information of specific AS events involved in each AS signature and final prognostic model.

AS events	Gene symbol	Splice type	Exons	From exon	To exon	*P*-value	Hazard ratio	95% Confidence interval	20-AS-event-based classifier
AA_CARM1_ID_47598	CARM1	AA	16.1:16.2	15	16.3	0.000498	0.269	0.128–0.563	
AA_ZBTB25_ID_27884	ZBTB25	AA	7.1	6	7.2	0.000596	0.3	0.151–0.596	
AA_GPBP1_ID_72126	GPBP1	AA	12.1	11	12.2	0.000859	0.325	0.168–0.629	
AA_ZNRF1_ID_37578	ZNRF1	AA	6.1	5	6.2	0.00228	2.8	1.45–5.43	^∗^
AA_DDX41_ID_74796	DDX41	AA	7.1	6	7.2	0.00388	0.392	0.207–0.74	
AA_CTDSP1_ID_57478	CTDSP1	AA	4.1	3	4.2	0.00441	0.386	0.201–0.744	
AD_OS9_ID_22701	OS9	AD	5.2:5.3	5.1	6	0.00235	2.7	1.42–5.13	^∗^
AD_HN1_ID_43371	HN1	AD	5.2	5.1	6	0.00427	0.388	0.203–0.743	
AD_THTPA_ID_26757	THTPA	AD	1.5	1.4	2	0.00511	0.377	0.19–0.746	^∗^
AD_NTMT1_ID_87866	NTMT1	AD	5.2	5.1	6	0.00556	0.395	0.205–0.761	^∗^
AD_MGME1_ID_58753	MGME1	AD	2.2	2.1	3	0.00625	0.415	0.221–0.779	^∗^
AD_SEC31A_ID_69735	SEC31A	AD	10.2	10.1	11	0.00879	0.424	0.223–0.806	
AP_SEC22A_ID_66462	SEC22A	AP	2.1			0.000205	0.258	0.126–0.527	
AP_ALG3_ID_67851	ALG3	AP	2.1			0.00032	0.277	0.138–0.558	^∗^
AP_PACS2_ID_29630	PACS2	AP	2			0.000733	3.2	1.63–6.28	
AP_ECE2_ID_67857	ECE2	AP	4			0.00103	4.45	1.83–10.8	^∗^
AP_HSP90AB1_ID_76378	HSP90AB1	AP	2			0.00182	0.347	0.179–0.675	
AT_MAGT1_ID_89535	MAGT1	AT	11			0.000186	3.74	1.87–7.47	^∗^
AT_RCBTB1_ID_25898	RCBTB1	AT	14			0.000294	3.46	1.77–6.78	^∗^
AT_SIN3B_ID_48214	SIN3B	AT	8.2			0.000508	3.41	1.71–6.8	
AT_SARNP_ID_22252	SARNP	AT	10.2			0.00053	0.297	0.149–0.59	
AT_ZNF675_ID_48822	ZNF675	AT	4			0.000571	0.275	0.132–0.574	
AT_STOX2_ID_71289	STOX2	AT	4			0.00067	5.28	2.02–13.8	^∗^
AT_NIPAL3_ID_1110	NIPAL3	AT	9			0.000837	0.33	0.172–0.633	^∗^
ES_NDUFA12_ID_23737	NDUFA12	ES	3	2.2	5.1	0.000239	0.248	0.118–0.522	^∗^
ES_UBR4_ID_880	UBR4	ES	105	104	106	0.00041	0.271	0.131–0.559	^∗^
ES_COPS3_ID_39468	COPS3	ES	9	8	10	0.000502	0.283	0.139–0.576	^∗^
ES_ABCE1_ID_70753	ABCE1	ES	14	13	15	0.000691	0.312	0.159–0.612	
ES_CCNI_ID_69628	CCNI	ES	2	1	3	0.000697	0.316	0.162–0.615	
ES_RPAP1_ID_30096	RPAP1	ES	22.2:23.1	22.1	23.2	0.000787	0.3	0.149–0.606	^∗^
ME_HLCS_ID_96019	HLCS	ME	4| 5	3	6	0.00111	0.338	0.176–0.649	^∗^
RI_RBM48_ID_80441	RBM48	RI	4.2	4.1	4.3	0.000128	0.235	0.112–0.493	
RI_RBM6_ID_64936	RBM6	RI	14.2	14.1	14.3	0.000144	0.263	0.132–0.524	
RI_RPAP1_ID_30095	RPAP1	RI	23.3	23.2	23.4	0.000599	0.279	0.135–0.578	^∗^
RI_METTL17_ID_26476	METTL17	RI	9.2	9.1	9.3	0.000622	0.298	0.149–0.596	
RI_POMGNT1_ID_2787	POMGNT1	RI	12.2	12.1	12.3	0.00122	0.333	0.171–0.648	^∗^
RI_TRABD_ID_62792	TRABD	RI	11.2	11.1	11.3	0.0015	2.84	1.49–5.42	^∗^
RI_WDR6_ID_64794	WDR6	RI	4.5	4.4	4.6	0.00155	0.34	0.174–0.663	^∗^
RI_FASTK_ID_82335	FASTK	RI	5.4	5.3	5.5	0.00194	0.334	0.167–0.668	
RI_NAA38_ID_81579	NAA38	RI	1.2	1.1	1.3	0.00199	0.349	0.179–0.68	

^∗^*Indicates AS events included in the final prognostic model.*

**Figure 7:**
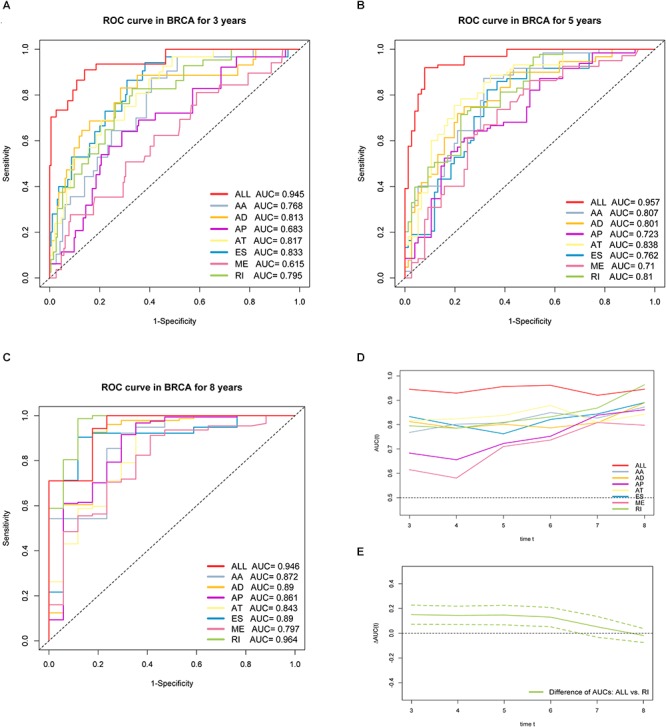
ROC curves with calculated AUCs of prognostic signatures built by either one type or all seven AS types in BRCA cohort for risk prediction in 3 years. **(A)** 5 years **(B)** and 8 years **(C)**, respectively. **(D)** The curves of time-dependent AUCs versus time (3–8 years) of either each or final signature: AUC(t) versus t. **(E)** The curve of the difference of time-dependent AUCs between final and RI signature over time (3–8 years): 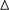
AUC(t) versus t. The color dashed bands indicated the pointwise 95% confidence intervals of estimated AUCs difference. The dashed line evaluates whether the difference of two estimated AUCs at each timepoint is statistical significance.

Furthermore, ROC curves estimated from 3 to 8 years survival were applied to compare the efficiency among different AS signatures. As presented in Figure 7, the AUCs were obviously varied among different splice type models. It is confirmed that the final prognostic AS signature exhibited the most robust and valuable predictive efficiency than other signatures built on a specific AS type, with AUCs keeping above 0.9 over time (Figure 7D). Although it seems that the 8 years AUC of RI were even higher than that of final signature, the difference was not statistically significant (*p* = 0.532), which might be due to the limited following-up data. On the contrary, the AUCs of final model were significantly highest compared with the others nearly up to 7 years (Figure 7E and Supplementary Figure S2). Thus, the final AS signature is non-inferiority in performance and exhibits much more prognostic efficiency.

Besides, in order to investigate the independent prognostic efficacy of the risk signature in stratified BRCA cohorts, patients were classified by available clinicopathological characteristics, including age, histological subtype, pathologic stage, Her-2 status, ER status, PR status, T stage, and N stage. And the stratification Cox analyses convinced that the low-risk subgroup had significantly longer OS than the high-risk group in all cohorts (Supplementary Figure S3). These results suggest that classification of the final AS signature can maintain its survival impact on precisely identify patients with poor prognosis, irrespective of clinical parameters.

### Gene Set Enrichment Analysis (GSEA) for 20 AS Event-Based Classifier

The strong stratification power of 20-AS-event-based signature in predicting prognosis, especially long-term survival probability of BRCA patients, might be largely attributed to their indispensable roles in tumor initiation and progression. Therefore, GSEA was further performed to identify their underlying splicing related mechanisms. NF-kappaB pathway, gradually recognized for its vital role involved in angiogenesis, epithelial-mesenchymal transition, anti-apoptosis, and tumor metastasis, was found enriched in the high-risk subgroup consistent with results of functional enrichment and GSVA mentioned above. Furthermore, several cancer related pathways were also significantly enriched in high-risk subgroup, including “signaling by Wnt,” “mismatch repair,” “p53 dependent G1 DNA damage response,” “SCF β-TRCP mediated degradation of EMI1,” “SCFSKP2 mediated degradation of P27/P21,” “FOXMI pathway,” “BARD1 pathway,” and “ARF6 downstream pathway,” which were already proved involved in the oncogenesis and progression of BRCA. In summary, GSEA results corroborated potential splicing-associated mechanisms and contributed to further reveal the pathogenesis and progression of BRCA (Supplementary Figure S4).

### Development and Apparent Performance of AS-Clinicopathologic Nomogram

**Figure 8:**
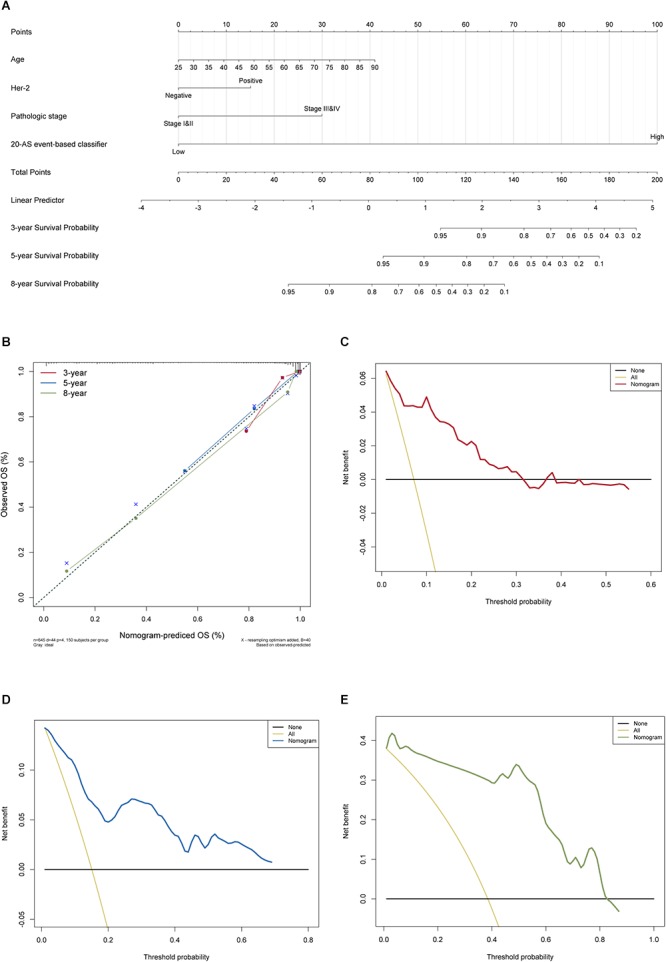
The AS-clinicopathologic nomogram for prediction on survival probability in patients with BRCA. **(A)** Development of AS-clinicopathologic nomogram for predicting 3-, 5-, and 8-years OS for BRCA patients, with the final AS signature, age, pathological stage and Her-2 status incorporated. **(B)** Calibration plot of the AS-clinicopathologic nomogram in terms of agreement between nomogram-predicted and observed 3-, 5-, and 8-years outcomes in BRCA cohort. The actual performances of our model are shown by red, blue and green lines. And the dashed line of 45° represents the ideal performance. **(C–E)** Decision curve analyses of the AS-clinicopathologic nomogram for 3-, 5-, and 8-years risk in BRCA cohort. The yellow line represents the net benefit of treat-all scheme varying with threshold probability, while the black line represents the net benefit of treat-no scheme. The net benefits by using our nomogram for predicting 3-, 5-, and 8-years OS are displayed with red, blue, and green lines, respectively.

The results of univariate Cox analysis on clinicopathologic characteristics are listed in Table 2, which showed that age, ER status, regional lymph nodes involvement, pathological stage and N stage were independent prognostic factors in BRCA cohort. With backward stepwise selection on optimizing AIC applied, a total of 4 variables including 20-AS-event based signature, age, pathological stage and Her-2 status were final incorporated in subsequent nomogram construction, even though Her-2 status was not identified as a prognostic factor in univariate Cox analysis of this cohort probably due to the limited data (Figure 8A). And the calibration cure of this nomogram for the probability of survival at 3, 5, or 8 years demonstrated good agreement between prediction and actual observation (Figure 8B). And the C-index for OS prediction was 0.883 (95% CI, 0.844–0.921). The DCA for this nomogram for 3, 5, or 8 years is also present, respectively (Figure 8C–E). The results showed more clinical usefulness of the constructed nomogram in predicting long-term survival probability, especially in 5 and 8 years, which meant that if the threshold probability of a patient or doctor was less than 80%, using this nomogram to predict prognosis in 5 or 8 years added more benefit than either the treat-none scheme or treat-all scheme. However, the 3 years DCA indicated a limited range of threshold probability only up to nearly 30%.

**Table 2:** Univariate cox proportional hazard analysis of clinicopathologic variable influence in BRCA cohort.

Features	Events (*N* = 645)	Crude HR (95% CI)	Log-rank *P*
Age			0.0414
<60	370		
≥60	275	1.8635 (1.025–3.389)	
Histological type			0.1^∗^
Infiltrating ductal carcinoma	456	1	
Infiltrating lobular carcinoma	130	0.3708 (0.1302–1.056)	
Other	59	1.1786 (0.4903–2.833)	
PR			0.204
Negative	210	1	
Positive	435	0.6737 (0.3664–1.239)	
ER			0.0362^∗^
Negative	144	1	
Positive	501	0.5152 (0.277–0.9582)	
Her2			0.267
Negative	538	1	
Positive	107	1.4987 (0.734–3.06)	
Positive lymph nodes			0.002^∗^
0	329	1	
1∼3	210	1.590 (0.7813–3.234)	
> 3	106	3.511 (1.6670–7.397)	
Pathology stage			0.0005^∗^
Stage I and II	501	1
Stage III and IV	144	2.889 (1.551–5.381)	
T			0.6
T1	186	1	
T2	362	0.8604 (0.4333–1.708)	
T3 and 4	97	1.3287 (0.5479–3.222)	
N			0.0005^∗^
N0	318	1	
N1	220	1.430 (0.7152–2.859)	
N2	68	1.982 (0.7659–5.126)	
N3	39	7.042 (2.5056–19.791)	
20-AS event-based classifier			0.0000000000008^∗^
Low	323	1	
High	322	58.910 (8.052–431)	

*ER, estrogen receptor; PR, progesterone receptor; Her-2, human epidermal growth factor 2.*

### Construction of Potential SF-AS Regulatory Network

**Figure 9:**
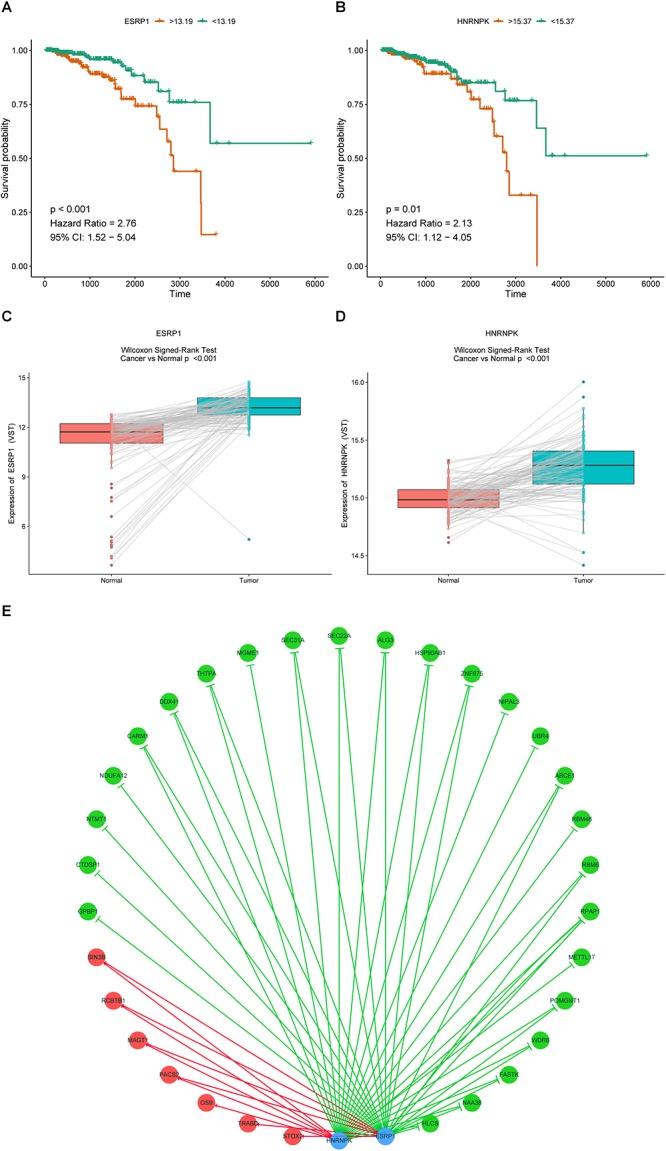
Prognostic SFs and the splicing correlation network in BRCA. **(A,B)** Survival curves of prognostic SFs. **(C,D)** The difference of expression values of prognostic SFs between primary BRCA and paired adjacent normal tissues. **(E)** Construction of SF-AS regulatory network. Expression values of survival-associated SFs (blue dots) were positively (red line) or negatively (green line) correlated with PSI values of AS events included in all types of AS signatures. The protective AS events are indicated by green dots while risky AS events are indicated by red dots.

It is widely accepted that globally dysregulated AS events were orchestrated by a limited number of SFs. With cross-reference of RNA sequencing profiling and corresponding clinical data of BRCA cohort, 2 out of 67 collected SFs were identified associated with OS, including ESRP1 (*P* < 0.001, HR = 2.76, 95% CI, 1.52–5.04) and HNRNPK (*P* = 0.01, HR = 2.13, 95% CI, 1.12–4.05), with the optimal cut points to classify patients into low- and high-risk groups (Figure 9A,B). Wilcoxon matched-pairs signed-rank tests were also conducted between cancer and matched normal samples to confirm the significantly differential expression of these two SFs (Figure 9C,D). Then Spearman correlation analysis was performed to estimate the prospective correlation between the expression level of ESRP1 and HNRNPK and the PSI scores of 40 OS-related AS events from prognostic signature in each AS type. Statistically, a total of 33 splicing events were correlated with ESRP1 or HNRNPK (blue dots), comprised of 7 events (HR > 1, red dots) indicating poor prognosis and 26 favorable events (HR < 1, green dots) highlighting better clinical outcomes. As was shown in Figure 9E, all favorable prognosis AS events were downregulated by ESRP1 or HNRNPK, whereas all adverse prognosis ones were upregulated by them, which were accordant with their expression levels and corresponding biological effects.

## Discussion

Owning to the rapid development of the high throughput sequencing technologies over the last decades, great success has been gained in the research of potential significance of AS profiling in BRCA biology. Evidences proved that specific dysregulation of splicing played critical roles in BRCA initiation, progression and metastasis. For instance, CD44v isoform, an alternative splicing variant of CD44 containing v8–v10 exons, was convinced to drive tumor progression and metastasis by promoting BRCA stemness via activating the PDGFRβ/Stat3 cascade and PFKFB4-mediated glucose metabolism despite tumor-suppressing genetic origin (Gao et al., 2018; Zhang et al., 2019). Similarly, Exon Skipped (ES) occurred in FLNB has been revealed to be associated with promoting epithelial-mesenchymal transition (EMT) in basal-like BRCA via releasing of FOXC1 transcription factor and decreasing FLNB nuclear localization (Li et al., 2018a). In this study, ES events of FLNB was significantly associated with OS with modified cutoff value while the ES of CD44 (also known as CD44v) was statistically significant at median cut (Supplementary Figure S5). These studies convinced that our present work could be largely consistent with previous results. Even though previous studies have mainly focus on monogenic isoforms, they provided a glimpse of AS in BRCA and comprehensive profiling of AS signatures in BRCA might further uncover its indispensable biological roles.

The tumorigenesis of BRCA is a complex regulatory network, integrating multiple biomarkers into an aggregated model could add more prognostic efficiency compared with intuitional clinical indicators alone. Over the past decade significant efforts have been made to integrate genomic-wide prognostic biomarkers for improvement of prognosis and diagnosis of BRCA. For instance, Li et al. (2018b) identified and validated a five-lncRNA set to predict risk of tumor recurrence through analysis of re-annotated lncRNA profiling in 891 BRCA samples from Gene Expression Omnibus (GEO). Meng et al. (2014) identified a four-lncRNA signature through analysis of 4 lncRNA GEO datasets to predict OS for BRCA patient using the random survival forest algorithm. Besides, Cardoso et al. (2016) validated the clinical usefulness of the addition of the 70-gene signature to standard clinical-pathological criteria in selecting patients for adjuvant chemotherapy by enrolling 6693 women with early-stage BRCA. Nevertheless, main focuses of these research were restricted to the transcriptome-level analysis to mining the prognostic mRNA, lncRNA, or miRNA signature. The prognostic value of AS was largely considered as the untapped potential. Recently, Shen et al. (2016) has developed a novel statistical model named SURVIV, with which the associations between alternative isoform variations of exon-skipping type and patient survival time were assessed in TCGA invasive ductal carcinoma cohort. Algorithmically, the established SURVIV model was based on exon-inclusion level of corresponding exon site for an individual ES event. However, the method generalization to model other types of AS is considerable for further validation. Additionally, owning to the distinct splicing pattern of seven types of AS, this quantification for splicing events is too abstract to follow. In our study, the PSI value, a common and intuitive ratio for quantifying splicing events, was introduced to make it possible for integrative analysis of seven types of AS events within cross-tumor or tumor-normal splice variations. To the best of our knowledge, the current study is the first attempt to perform a comprehensive understanding and identification of OS-oriented AS signature in BRCA tissues. As a result, a total of 3071 alternative splicing events from 2075 parent genes were significantly associated with OS in BRCA. Meanwhile, the final prognostic predictor built by combination of all available AS types showed a robust and significant improved performance with AUCs maintaining above 0.9, comparing to all the predictors built with only one type of AS, which suggested that AS hold great potential significance in application of prognosis prediction for BRCA patients.

Furthermore, we postulated that the inclusive model combining the AS signature and important clinical parameters may achieve a more reliable and favorable prediction efficacy for predicting survival probability. Indeed, prognostic nomogram integrated with age, Her-2, pathological stage and 20 AS signature was recommended for evaluating individualized survival risk, with satisfactory discrimination achieved (C-index, 0.883, 95% CI, 0.844–0.921). However, the clinical consequences of a particular level of performance, discrimination or degree of miscalibration cannot be merely justified by AUC value of ROC curve, C-index or calibration curve of prognostic model. Thus, we applied DCA to assess if nomogram assisted decisions improve patient outcomes, which is much more practical and high-efficiency than multi-institutional prospective validation. This novel statistical approach derived a graphical analysis of the net-benefit against a range of threshold probabilities (Threshold probability is defined as a cutoff where the expected benefit of treatment equals expected harm of avoiding treatment). Notably, the 5 and 8 years decision curves exhibited much more tolerance with threshold probabilities up to 80%, which means that using the AS-clinicopathologic nomogram to predict long-term survival probabilities adds more benefit than either all of patients were treated or none of them were treated.

Besides, we attempted to investigate the potential mechanism of prognostic AS events in BRCA. Notably, the CC aspect of GO analysis results in our present work indicated that modifying protein feature through variation on these genes’ transcript architecture (alternative splicing) can be mediated by mitochondria associated pathways, which was regarded as the key regulator of apoptosis by triggering complex cell-death process (Chen et al., 2016). The activities of Cell adhesion molecules (Figure 3B) formed glue to precede focal adhesion formation, and altered focal adhesion dynamics is associated with cancers (Maziveyi and Alahari, 2017). The functional enrichment analysis also indicated several significant interfered pathways, such as ubiquitin mediated proteolysis, spliceosome-related pathways and NF-KappaB signaling pathway, which were in accordance with preliminary studies concerning the genome-wide investigation of AS in gastrointestinal adenocarcinomas and colorectal cancer, respectively (Lin et al., 2018; Xiong et al., 2018). Therefore, we would venture to guess that cancer-related outcome resulted from AS alteration may be disturbed via some shared cancer pathways. In addition, the integrative results of GO, KEGG, and GSVA revealed some cancer-specific pathways, such as cell adhesion, HIF-1 signaling pathway and FAS signaling pathway, supporting the reliability and accuracy of our present *in silico* analyses.

Moreover, GSEA analysis for ranked mRNA profiling in BRCA cohort analyzed the differential pathways enriched in high-risk group versus low-risk one as stratified by the 20 AS-event signature. Similar to previous studies, GSEA analysis also provided a better understanding of underlying molecular mechanisms involved in BRCA prognosis. Results showed that 20 AS events derived from 19 parent genes were involved in several cancer-related pathways including Wnt signal pathway, activation of NF-kappa B, p53 independent DNA damage repair response in mitosis pathway, which were proven the crucial roles in regulation of BRCA oncogenesis and progression. Therefore, the GSEA results also provided valuable clue to BRCA-related biological pathways through 20-AS-event signature, contributing to tumor progression such as proliferation, invasiveness, and metastasis of BRCA. So far, the exact biological effects of these AS events have not been validated, and it was worthwhile deciphering the underlying mechanisms for search of valuable therapeutic targets for BRCA treatment.

SFs were important regulatory dominators of AS events by recognizing and binding to cis-regulatory elements of pre-mRNA and then influencing exon selection and splicing site choice. In our study, we constructed a potential SFs-ASs correlation network between proposed prognostic SFs and most significantly survival-associated AS events, and overview of the network revealed obvious trends that overexpression of ESRP1 and HNRNPK were negatively correlated with favorable prognosis AS events and positively correlated with adverse prognosis AS events, which was in accordance with the results of survival analysis. Meanwhile, previous research on splicing regulation of SFs was also reflected in the exploration of AS signature in BRCA. Horiguchi et al. (2012) revealed that downregulation of epithelial splicing regulatory protein 2 (ESR2) might get related to the TGF-β-induced EMT and progression of BRCAs by dysregulating of alternative splicing. Shapiro et al. (2011) also confirmed the overexpression of epithelial-specific splicing factor ESRP1 and depletion of RBFOX2 in mesenchymal BRCA cells could drive critical EMT-associated phenotypic changes in cell morphology and motility through regulating functional EMT-associated AS signature. Besides, Yae et al. (2012) reported that knockdown of ESRP1 in CD44 variant isoform-expressing (CD44v +) subpopulation of 4T1 BRCA cells resulted in an isoform switch to CD44 standard (CD44s), downregulating cell surface expression of cystine transporter (xCT) and depressing the lung colonization for further prevention of metastasis. In addition, Brown et al. (2011) also suggested ESRP1 controlled the transformation of CD44 to CD44s isoform, which regulated the EMT phenotype and contributed to BRCA progression by activating Akt signaling pathway. HNRNPK, a membership of hnRNP family, which was recognized for binding to splicing enhancers or silencers and acting as splicing activators or suppressors, had been proved to mediate tumorigenesis through splicing regulation of MRPL33-L isoform, promoting cancer cell growth and repressing apoptosis (Leopoldino et al., 2007; Liu et al., 2018). Additionally, Tyson-Capper and Gautrey (2018) reported the HNRNPK could switch the Myeloid cell leukemia-1 (Mcl-1) toward proapoptotic Mcl-1S isoform in two BRCA cell lines, and its overexpression was related to poor prognosis and drug resistance for BRCA. However, few studies have reported the actual regulation mechanism between these two prognostic SFs and AS signature identified in our study, and further elucidation with functional experiments is urgently needed.

Although our model performs well in BRCA prognosis prediction, there are inevitably several limitations in current study that still need clarification. First, patients enrolled in our cohort were exclusively from a single database with relatively small sample size, and no another independent cohort, especially prospective one, is available to validate that nomogram being proposed here is reproducible. Second, the clinicopathological characteristics analyzed in our cohort are not comprehensive due to limited released publicly data, which might serve to bias our results. Nevertheless, randomized clinical trials are warranted to verify our present silico analysis in the future.

## Conclusion

In conclusion, we have presented a systematic approach for prognostic splicing variants in BRCA and constructed a well-performed nomogram combing clinicopathologic variables with 20-AS-event-based signature. What’s more, the candidate AS events identified in our study, especially the 20 AS events taken into the final signature, which perhaps consisted of the most valuable AS events in deciphering the underlying mechanism in oncogenesis and pathogenesis of BRCA, possessed great potential in clinical implications as molecular diagnostic biomarkers and therapeutic targets for BRCA patients.

## Data Availability

Publicly available datasets were analyzed in this study. This data can be found here: https://cancergenome.nih.gov/.

## Author Contributions

QY conceived and directed the project. DZ designed the study and analyzed the data. DZ and YD wrote the manuscript. JC reviewed the data. All authors have read and approved the final manuscript for publication.

## Conflict of Interest Statement

The authors declare that the research was conducted in the absence of any commercial or financial relationships that could be construed as a potential conflict of interest.

## Footnotes

1 https://portal.gdc.cancer.gov/

2 www.broadinstitute.org

3 http://www.introni.it/splicing.html

## Supplementary Material

FIGURE S1 Illustration for the splicing pattern of 20 particular AS events included in final 20-AS events-based classifier. The blue box indicates the exon while gray box indicates the intron. The specific AS events are marked with red lines. The parent genes and splicing types are presented on the left side.

FIGURE S2 The curve of the difference of time-dependent AUCs between final and other AS signature over time (3–8 years): AUC(t)versus t. The dashed bands indicated the pointwise 95% confidence intervals of estimated AUCs difference. The dashed line evaluates whether the difference of two estimated AUCs at each timepoint is statistical significance. **(A–F)** The curve of the difference of time-dependent AUCs between final and other AS signatures, including AA **(A)**, ES **(B)**, ME **(C)**, AP **(D)**, AD **(E)**, and AT **(F)**, respectively.

FIGURE S3 Application of the final AS signature in stratified BRCA cohorts. **(A–H)** Stratified Cox analysis of overall survival between high- and low-risk patients classified by different clinicopathological characteristics, including age **(A)**, histological subtype **(B)**, pathologic stage **(C)**, Her-2 status **(D)**, ER status **(E)**, PR status **(F)**, T stage **(G)**, and N stage **(H)**.

FIGURE S4 GSEA delineates biological processes and pathways correlated with the final 20 AS event-based classifier using gene sets of “c2.cp.v6.1.symbols” download from MSigDB. The BRCA cohort was divided into low- and high-risk subgroups. The running was conducted with 1000 permutations. GSEA validated enhanced activity of **(A)** “signaling by Wnt,” **(B)** “p53 dependent G1 DNA damage response,” **(C)** “SCF β-TRCP mediated degradation of EMI1,” **(D)** “SCFSKP2 mediated degradation of P27/P21,” **(E)** “regulation of ornithine decarboxylase odc,” **(F)** “mismatch repair,” **(G)** “activation of NF-KappaB in B cells,” **(H)** “FOXMI pathway,” **(I)** “BARD1 pathway,” and **(J)** “ARF6 downstream pathway.”

FIGURE S5 Kaplan-Meier plots of previous reported AS events in BRCA. **(A)** ES of CD44 (CD44v) were significantly survival associated with OS at median cut. **(B)** ES events of FLNB was significantly associated with OS with modified cutoff value.

TABLE S1 Collection of 67 human splicing factors from the SpliceAid 2 database.

TABLE S2 Detailed enrichment results of GO terms and KEGG pathways.

## Abbreviations

AA: Alternate Acceptor site
AD: Alternate Donor site
AP: Alternate Promote
AS: Alternative splicing
AT: Alternate Terminator
AUC: Area under the curve
BH: Benjamini and Hochberg
BP: Biological process
BRCA: Breast cancer
CC: cellular component
DCA: Decision curve analysis
ES: Exon Skip
FDR: False discovery rate
GO: Gene ontology
GSEA: Gene set enrichment analysis
GSVA: Gene Set Variation Analysis
HR: Hazard ratios
KEGG: Kyoto Encyclopedia of Genes and Genomes
ME: Mutually Exclusive Exons
MF: Molecular function
MSigDB: Molecular Signatures Database
OS: overall survival
PSI: Percent Spliced In
RI: Retained Intron
ROC: receiver-operator characteristic
SFs: Splicing factors
TCGA: the Cancer Genome Atlas.
