# Supplementary figures and images for: Identification of Prognostic Alternative Splicing Signature in Breast Carcinoma
Source: Front Genet. 2019 Mar 28;10:278. doi: 10.3389/fgene.2019.00278 (PMC6448481; doi:10.3389/fgene.2019.00278)

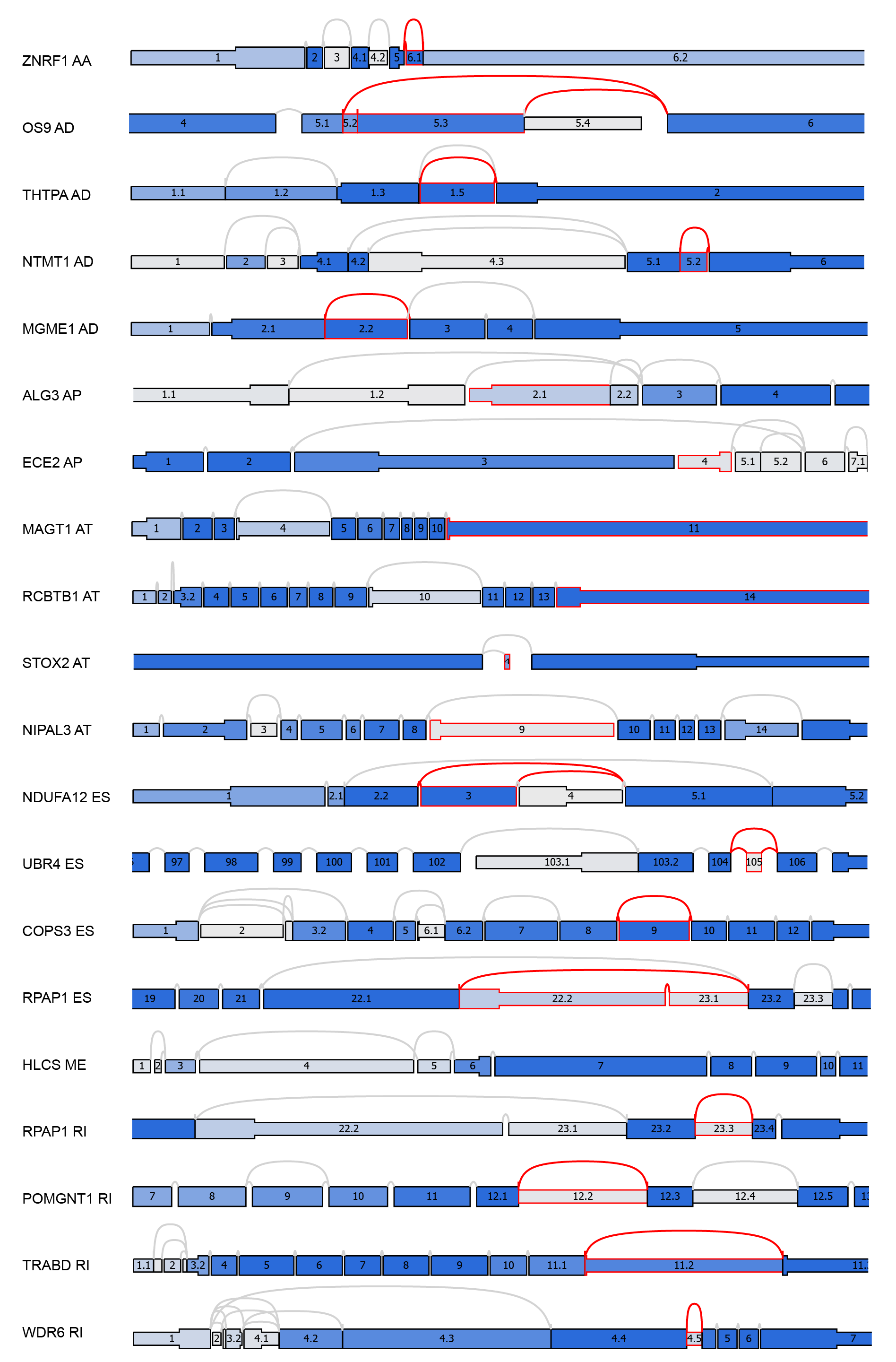

Supplement: FIGURE S1 — Illustration for the splicing pattern of 20 particular AS events included in final 20-AS events-based classifier. The blue box indicates the exon while gray box indicates the intron. The specific AS events are marked with red lines. The parent genes and splicing types are presented on the left side. [file Image_1.TIF]

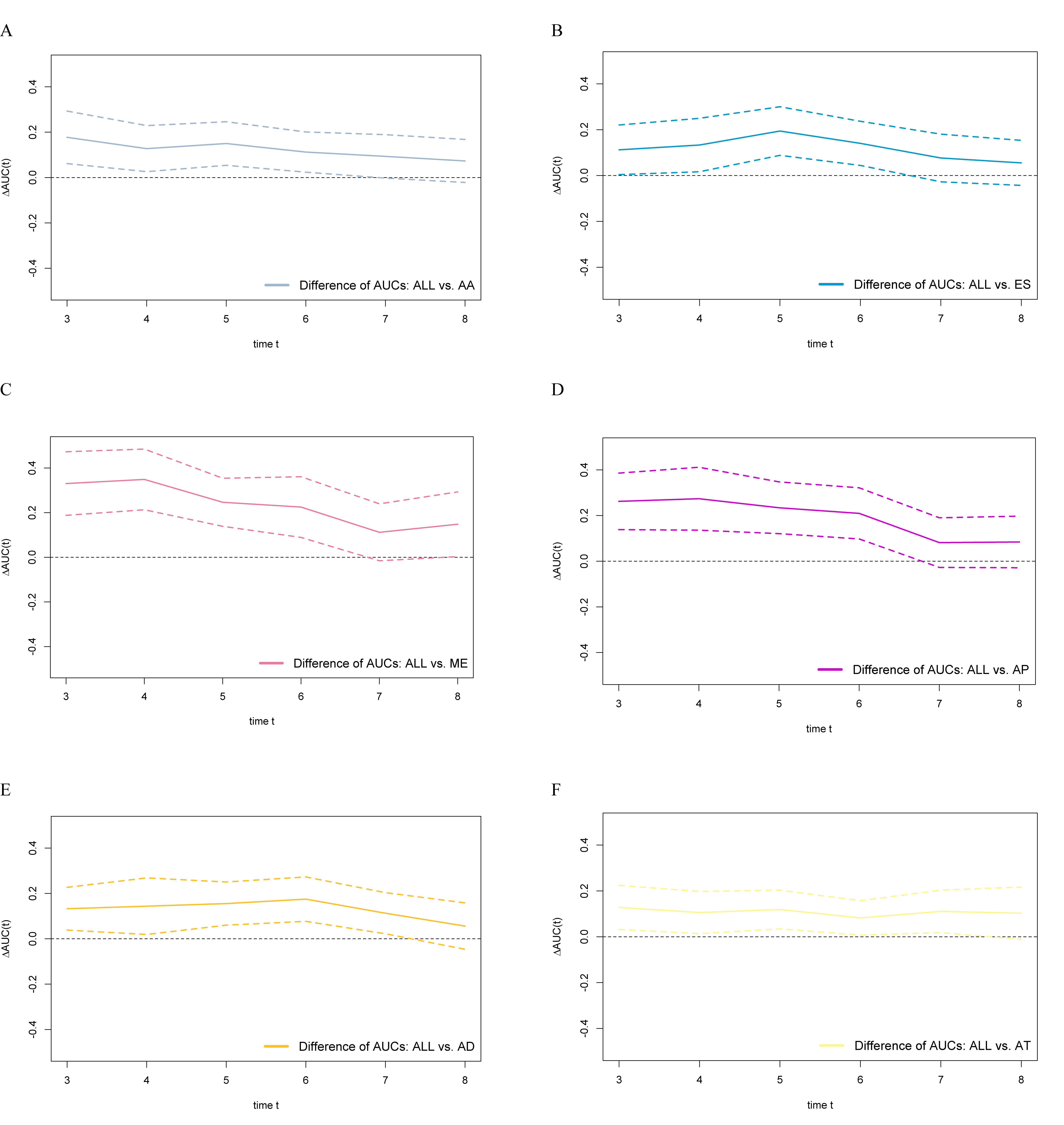

Supplement: FIGURE S2 — The curve of the difference of time-dependent AUCs between final and other AS signature over time (3–8 years): AUC(t)versus t. The dashed bands indicated the pointwise 95% confidence intervals of estimated AUCs difference. The dashed line evaluates whether the difference of two estimated AUCs at each timepoint is statistical significance. (A–F) The curve of the difference of time-dependent AUCs between final and other AS signatures, including AA (A), ES (B), ME (C), AP (D), AD (E), and AT (F), respectively. [file Image_2.TIF]

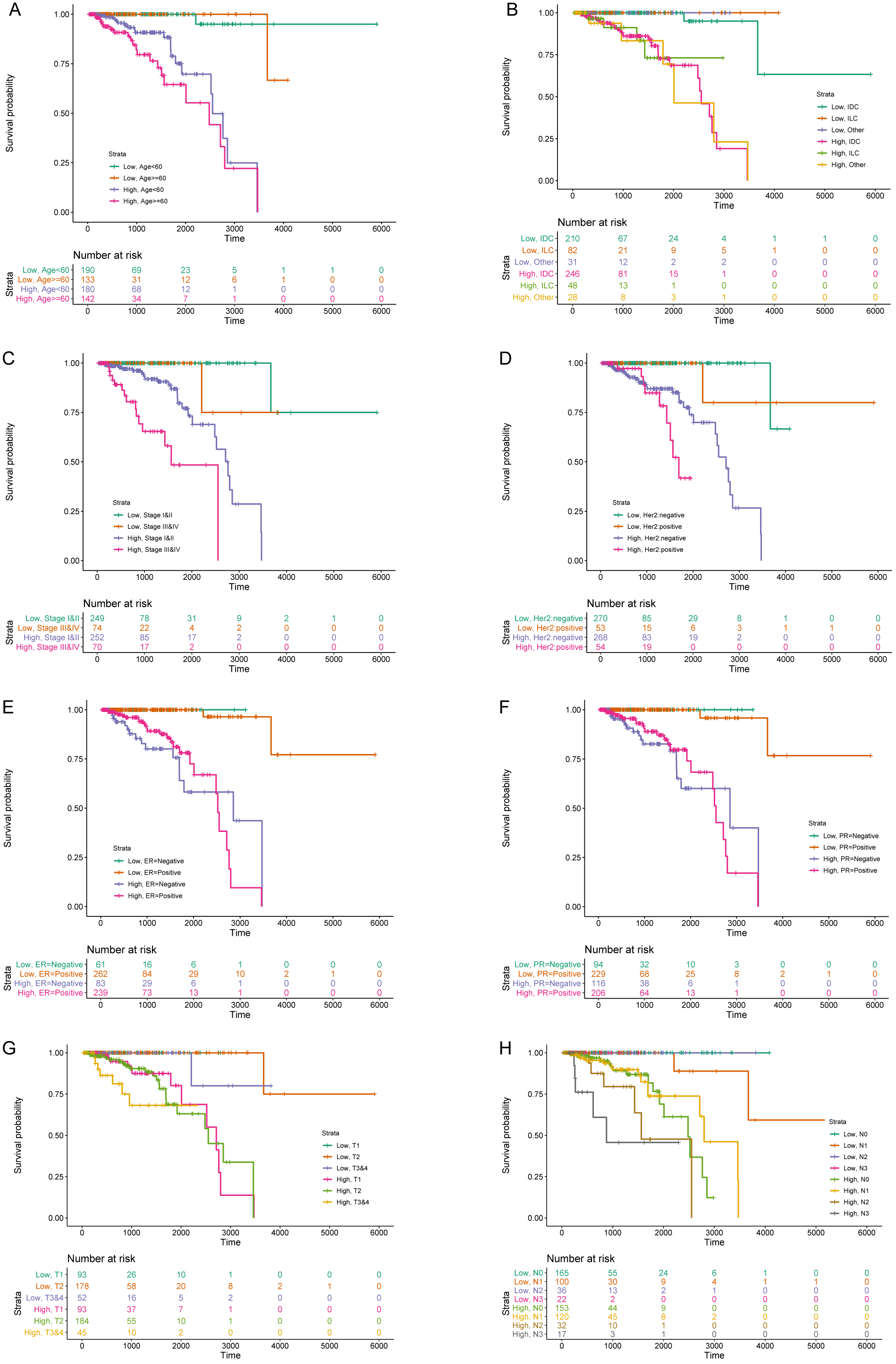

Supplement: FIGURE S3 — Application of the final AS signature in stratified BRCA cohorts. (A–H) Stratified Cox analysis of overall survival between high- and low-risk patients classified by different clinicopathological characteristics, including age (A), histological subtype (B), pathologic stage (C), Her-2 status (D), ER status (E), PR status (F), T stage (G), and N stage (H). [file Image_3.TIF]

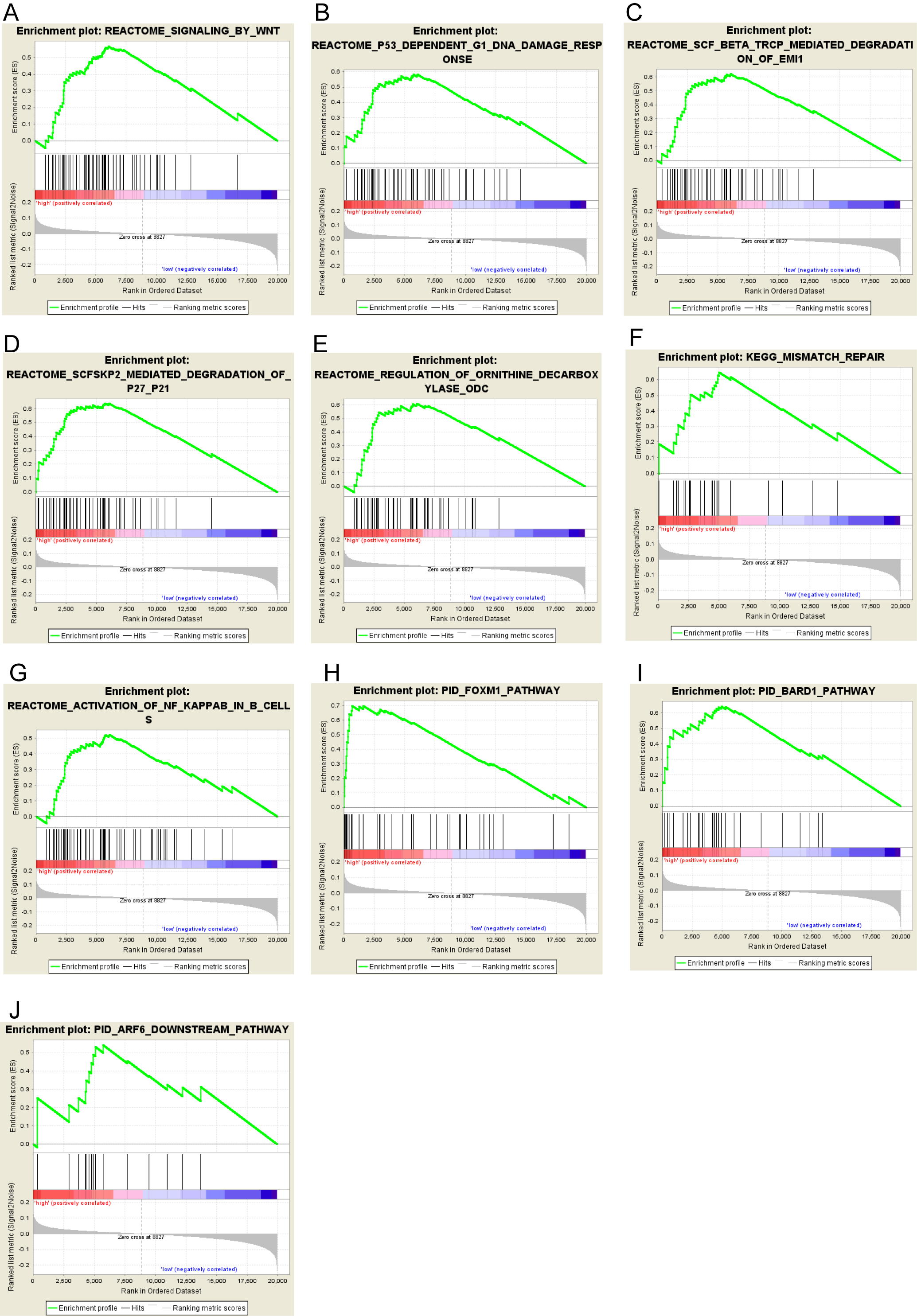

Supplement: FIGURE S4 — GSEA delineates biological processes and pathways correlated with the final 20 AS event-based classifier using gene sets of “c2.cp.v6.1.symbols” download from MSigDB. The BRCA cohort was divided into low- and high-risk subgroups. The running was conducted with 1000 permutations. GSEA validated enhanced activity of (A) “signaling by Wnt,” (B) “p53 dependent G1 DNA damage response,” (C) “SCF β-TRCP mediated degradation of EMI1,” (D) “SCFSKP2 mediated degradation of P27/P21,” (E) “regulation of ornithine decarboxylase odc,” (F) “mismatch repair,” (G) “activation of NF-KappaB in B cells,” (H) “FOXMI pathway,” (I) “BARD1 pathway,” and (J) “ARF6 downstream pathway.” [file Image_4.TIF]

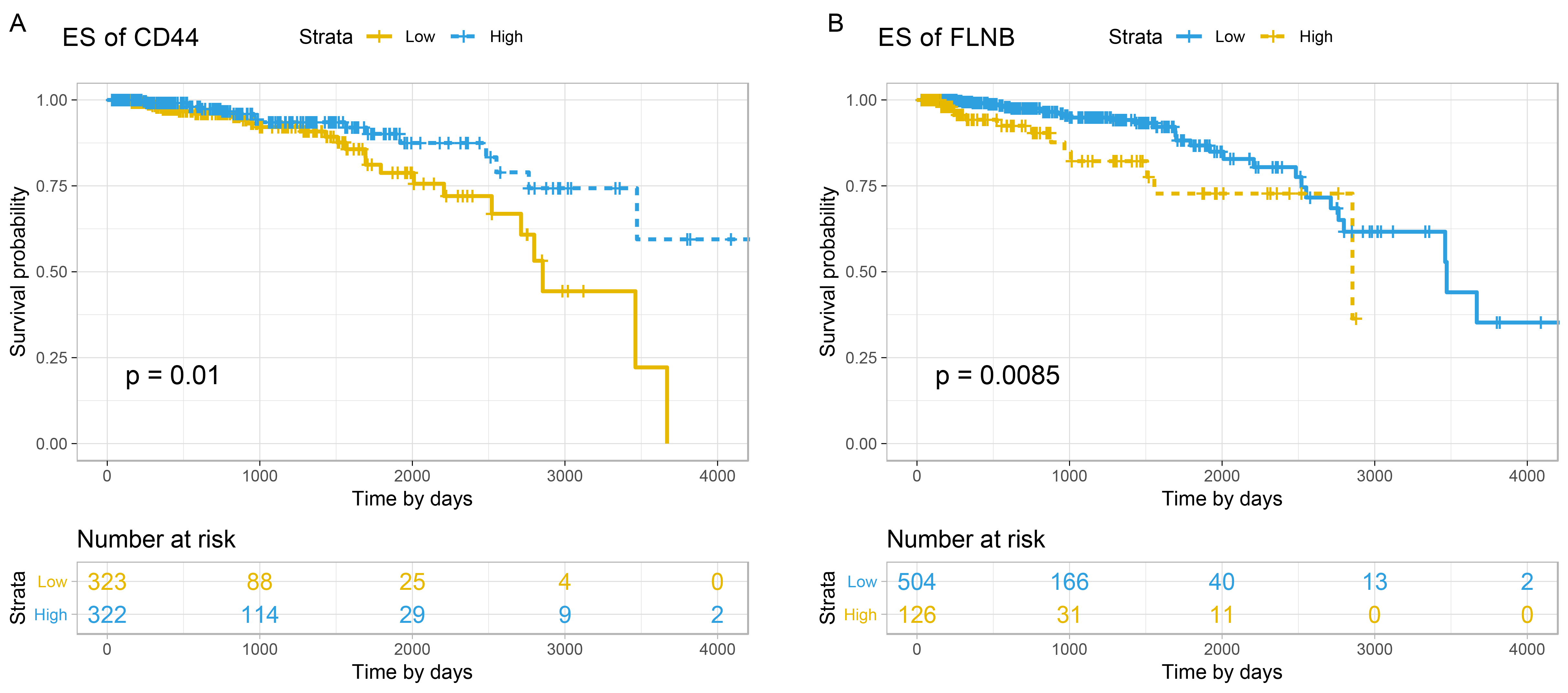

Supplement: FIGURE S5 — Kaplan-Meier plots of previous reported AS events in BRCA. (A) ES of CD44 (CD44v) were significantly survival associated with OS at median cut. (B) ES events of FLNB was significantly associated with OS with modified cutoff value. [file Image_5.TIF]
